# Strengthening Urban Informal Trading and Improving the Health of Vendors: An Integrated Management Model

**DOI:** 10.3390/ijerph20064836

**Published:** 2023-03-09

**Authors:** Maasago Mercy Sepadi, Vusumuzi Nkosi

**Affiliations:** 1 Department of Environmental Health, Faculty of Health Sciences, Doornfontein Campus, University of Johannesburg, Johannesburg 2094, South Africa; 2 Department of Environmental Health, Faculty of Sciences, Pretoria Campus, Tshwane University of Technology, Pretoria 0183, South Africa; 3 Environment and Health Research Unit, South African Medical Research Council, Johannesburg 2094, South Africa; 4 Faculty of Health Sciences, School of Health Systems and Public Health, University of Pretoria, Pretoria 0001, South Africa

**Keywords:** street vendors, working conditions, environmental health, street vendors, management models, South Africa, health, healthy workplaces

## Abstract

Context: Within the urban areas, especially the central business districts, informal trading is growing in large numbers, and the informal vendor’s health is also at risk. Despite various frameworks developed for this industry, there are few guidance and implementation strategies on how to accomplish better-managed informal trading, mostly one that entails better occupational settings. Objective: The goal of the proposed model is to improve the working conditions for informal vendors in South Africa by redesigning the current informal trading management approach, thus ensuring a healthy workplace. An evidence-based approach was used to inform the development of this model. Results: This paper outlines the current challenges of informal vendors in urban areas as per the quantitative health risk assessment study that was conducted in 16 markets amongst 617 informal food vendors in the inner city of Johannesburg, South Africa. The study investigated the respiratory health caused by air pollution and associated risk factors. Findings revealed a lack of infrastructure and higher exposure to air pollution, resulting in respiratory ill-health amongst outdoor vendors as compared to indoor vendors. The vendors were more exposed to particulate matter pollution in the spring and winter seasons as compared to the autumn and summer seasons. Furthermore, the upper respiratory symptoms were statistically significant to the type of work location (indoor/outdoor), type of cooking fuel, duration of work, frequency of hand hygiene, and wearing of protective equipment. An informal vendors’ integrated management model that encompasses a specific informal food vendor’s directorate was developed with five main components: the reviewing of informal vendors’ legislation, the restructuring of designated vending or trading sites, space allocation and occupancy, vendors‘ training and skills development, and the sustainability of vending sites and vendors’ health. Discussion and Conclusions: The status revealed the legislation fragmentation of the informal vendors’ activities. The goal of this informal vendors’ “healthy workplace management model” is to inform government responses to the current challenges of the informal vendors’ sector, as well as to guide policies and actions to reduce ill-health in the industry and to avoid disruptions to informal food supply chains, which are critical in the food sector. This model is explained well and documented for easier implementation in local governments. This paper contributes to the extant literature on street vendors and future management strategies of this trade.

## 1. Introduction

In the adoption of sustainable development goals (SDGs), governments worldwide commit to addressing poverty and the associated environmental and occupational health hazards experienced by people living under the conditions of poverty [1]. To help achieve health-related SDG targets, the government’s aim is to substantially reduce the morbidity and mortality resulting from hazardous chemicals and air, water, and soil pollution and contamination by 2030 [1]. According to Weigo (2014), obstacles faced by vendors include a lack of political support for illegal trade, police harassment, and the confiscation of items, as well as storage space [2]. When examining vendors’ activities, waste management was identified as a significant issue, along with crime, the lack of essential services (such as power and water sources at many stalls), and most vendors’ use of surrounding companies’ premises for their storage purposes [3]. A study conducted in Johannesburg found that there is a lack of infrastructure, and outdoor street trading is characterized by temporary structures for selling goods [4]. Hill et al.’s (2019) paper further argues that while current research acknowledges the economic impact of informal food vending, it neglects to look at potential improvements to the sector and ways to advance the knowledge and abilities of vendors [3]. Additionally, the lack of research and theory on street trading makes it difficult for authorities to adopt a new and inclusive strategy because managing traders in busy and diverse streets is more difficult than managing traders inside designated markets [5].

Cities can find a balance between the need to acknowledge other demands on public space [6] and the need to encourage informal livelihoods as per the SDGs [1]. The intended use of the proposed model is to promote a healthy workplace for informal vendors in South Africa (SA), by restructuring the current informal trading management strategy. This goal can be achieved by ensuring the universal management of informal vendors from inception or grass-root (registering as a vendor) to occupying well-designated markets and stalls and adhering to the legislations of interest. A healthy workplace is defined by the World Health Organisation (WHO) as “one in which workers and managers collaborate to use a continual improvement process to protect and promote the health, safety and well-being of workers and the sustainability of the workplace by considering the following, based on identified needs: health and safety concerns in the physical work environment; health, safety and well-being concerns in the psychosocial work environment including organization of work and workplace culture; personal health resources in the workplace; and ways of participating in the community to improve the health of workers, their families and other members of the community” [7]. Then, the Work-Life Initiative of the American National Institute for Occupational Safety and Health (NIOSH) envisions workplaces free of recognized hazards, with health-promoting and sustaining policies, programs, and practices; and employees with ready access to efficient programs and services that protect their health, safety, and well-being [8].

Moreover, informal food vendors are a key source of delivering affordable, accessible, and fresh food to communities and providing a very important source of income [2,9], and a healthy workplace management model supports the informal economy, as a vendor’s health is connected to productivity and income.

This model development was aimed at achieving the requirements of the Occupational Health and Safety (OHS) Act (no. 85 of 1993) [10] and the Regulations governing general hygiene requirements for food premises, the transport of food, and related matters (no. R638 of 22 June 2018) [11] which are essential for healthy occupational and food premises conditions. Moreover, it is integrating the OHS of informal vendors into the public health system, especially in local government.

## 2. Materials and Methods

**Figure 1:**
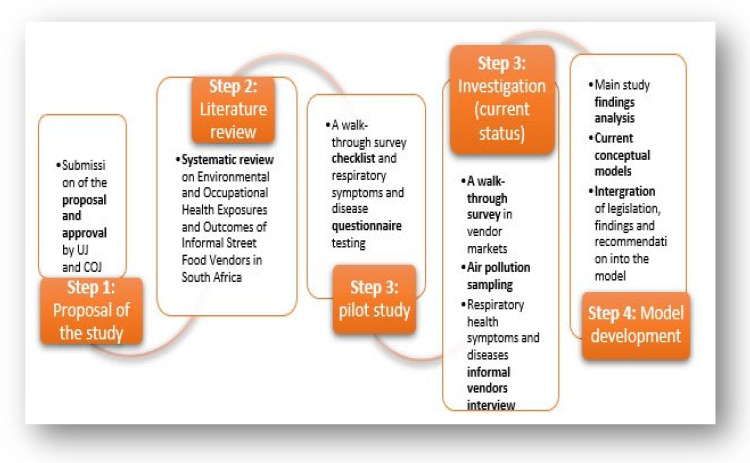
The process followed in developing the informal vendors’ integrated healthy workplace management model.

This evidence-based model was developed as one of the objectives of the study conducted in the inner City of Johannesburg (COJ) [12]. The current South African literature on the occupational settings of informal vending and conceptual models (and the findings of the recent study) was used for the development of this model (Figure 1), thus including the requirements of a healthy workplace model by the WHO. The majority of the findings in the recent study were published [12,13,14,15,16] excluding the content of the model and recommendations.

The recent study conducted in the inner City of Johannesburg followed the guidelines of the human health risk assessment (HHRA) [17] from the WHO by surveying the vendor workplace (markets and stall), identifying respiratory risk factors, measuring air pollutants and conducting interviews on the respiratory health symptoms and diseases, and a systematic review on the current environmental and occupational health statuses of informal vendors. The results were compared between the population of indoor and outdoor food vendors. Following a total sampling technique, the COJ’s inner-city health department’s (DOH) 2022 database (serving as the sampling source for this study and yielding 16 informal vendor marketplaces and 617 informal food vendors) was used as the sampling source for this study [12,16]. The total sampling of the limited population size of recorded informal food vendors that operated in approved markets within the study area made this type of sampling technique appropriate for the study. It was feasible to gain in-depth insights into the informal vending trade since the entire population was given a chance to participate.

The walkthrough survey focused on the respiratory risk factors of the trading markets, which include infrastructure and general hygiene. The close-ended questionnaire was adopted from the validated British Medical Research Council respiratory health questionnaire and supplemented with workplace-related questions [12]. This assessment was conducted through a face-to-face interview using the questionnaire with demographic information such as gender and age; employment factors such as duration of work in hours, days, and years; practices in the workplace; and self-reported respiratory symptoms and diseases within the last 12 months. Before the main study, the walkthrough survey checklist and respiratory health questionnaire were piloted in the South of Johannesburg amongst 100 informal food vendors [14] for validity and reliability and were then revised for the main study. SPSS descriptive analysis was used on these quantitative data, including estimates of the prevalence of diseases and symptoms of the respiratory system in proportion to population size reported as frequencies [18].

For the air pollution exposure assessment, five air pollutants were examined: particulate matter (PM_2.5_), Nitrogen Dioxide (NO_2_), Sulphur Dioxide (SO_2_), Carbon Monoxide (CO), and Carbon Dioxide (CO_2_) [15,16]. The Radiello passive sampler was used to detect NO_2_ and SO_2_, an EXTECH air quality monitor for CO and CO_2_, and the GilAir pump to collect samples for ambient PM_2.5_ concentrations. A total of 41 air samples were taken from the indoor and outdoor market: eight PM_2.5_ area samples, twenty-five personal PM_2.5_ samples, and two samples of NO_2_, SO_2_, CO, and CO_2_ [15,16]. The air sampling program was conducted on the same day in the winter season to establish each pollutant exposure during the respiratory health risk season according to the literature due to the weather conditions. To further understand the difference in PM exposures in different meteorological conditions, two samples from the indoor and outdoor stalls were collected in all yearly seasons. All air samples were over an 8 h work shift and analyzed in a South African National Accreditation System (SANAS)-approved laboratory. 

## 3. Results

### 3.1. Existing Literature and Concept Models

Prior to the main study, the authors conducted a systematic review of the environmental and occupational health exposures and outcomes of informal street food vendors in SA [14]. The studies in South Africa on environmental and occupational health risks and outcomes offered some evidence of the general health effects associated with the informal vendors’ industry. The findings of this review revealed the different types of street vending stalls which were in line with the recent international literature that highlights the various infrastructure issues that informal vendors encounter, such as a lack of adequate stall shelter. Informal vendors operating in the open public were indicated to be the most vulnerable to environmental hazards, particularly those operating from unprotected informal buildings or from stalls or workplaces that are not adequately weatherproofed.

The WHO’s Healthy Workplace Framework and Model (2010) avenues of influence, process, and core principles include components such as enterprise community involvement, psychological work environment, physical work environment, and personal health resources [7]. Bhubaneshwar was one of the first cities in India to recognize street vendors as an integral part of the city. The process of conceptualizing and designing a model of the vending area began through a partnership between the city government (public), other partners, and street vendors (community). This resulted in the setting up of aesthetic fixed kiosks in legal vending areas [19].

There have been limited models related to the health and safety of informal vendors or the informal vending trade in South Africa in recent years. A relatable study conducted by Hill et al., (2019) in the City of Cape Town, South Africa, developed a street food vendor model entailing four components: business, food and nutrition, hygiene, and vending carts [3]. Some elements of this study model are similar to the matters that the current study aims for, which are sanitary conditions at informal vendors’ workplaces. However, the study in Cape Town was based on food safety and the economic side of the trade, as it was aimed at a sustainable model for selling healthy and safe street food in the City of Cape Town, enabling street vendors to make a decent living and consumers to make healthy choices regarding food purchasing.

### 3.2. Summary of the Current Status of Informal Vendor Operations

Most vendors were cooking vendors (56%). In total, 73% worked longer than the recommended 8 h per day, 90% worked 6 to 7 days per week, and the majority had been working for between 6 and 10 years (42%). A total of 37% had access to market communal taps, followed by 35% with internal stall taps, with the majority being indoor vendors, and only 31% of the vendors practiced handwashing according to the WHO standards. The neighboring businesses or locations, such as parks, who were bringing water from home accounted for 27%, and only 1% stated they purchased water for their operations. A lack of water facilities for outdoor vendors was revealed, which may result in poor hand hygiene practices. The prevalence of upper respiratory symptoms was higher as compared to other respiratory health problems. Various risk factors identified amongst informal food vendors were found statistically significant with upper respiratory symptoms (cold, sore throat, and nasal congestion). This included work location (*p* < 0.001) and work duration in hours at *p* < 0.001. Work duration in days revealed the cold and sore throat symptom at *p* < 0.001 and nasal congestion at *p* = 0.001. Work duration in years revealed the cold symptom at *p* = 0.008, the sore throat symptom at *p* = 0.015, and nasal congestion at *p* = 0.005. The types of cooking fuel used were found at a *p* < 0.001. Cooking gas (45%) was the most common cooking fuel used, followed by electricity (13%), open fire, which accounted for 11%, and 7% used a combination of fuel types or more than one type of fuel. The informal vendor training was found with a cold at *p* = 0.003, sore throat (*p* = 0.021), and nasal congestion (*p* = 0.023). The frequency of hand hygiene practice and frequency of mask use was *p* < 0.001 [16]. 

The outdoor vendor market had greater concentrations of area air pollutants (PM_2.5_, SO_2_, NO_2_, and CO_2_) than the indoor market, with cooks being the most at risk. The PM exposure in comparison to meteorological conditions showed that spring and winter recorded higher exposures as compared to summer and autumn. These findings were consistent with several international studies [20,21,22]. Open-fire-usage markets have higher exposure levels, which increase the possibility of respiratory, cardiovascular, and reproductive system disorders [20,21,22,23]. This study’s findings also showed that there was the usage of incorrect respiratory protective equipment (RPE) (surgical and cloth masks) for the indicated air contaminants which may be ineffective in this trade [24]. Moreover, most vendors who received training reported only receiving it once a year. Seventy-five percent of the trained vendors came from indoor marketplaces, largely from the cooking category.

### 3.3. Integrating Findings into the Development of the Management Model

**Figure 2:**
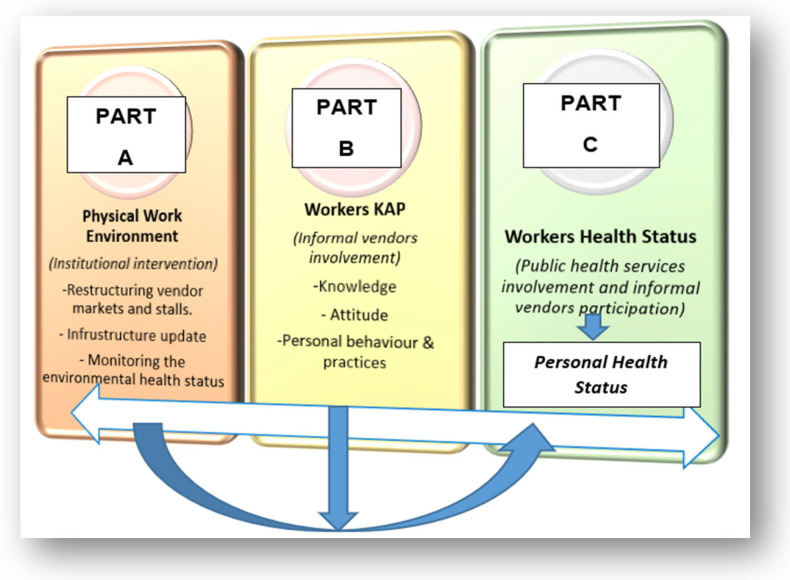
Informal vendors’ environmental and occupational health three-way framework.

The results of the current study showed that the efforts to improve the health of informal workers should consider the contexts in which they work in order to develop tailored interventions for specific subpopulations of informal workers [25]. The results of this study showed that the work environment and lack of infrastructure are associated with ill health, and the interventions in responding to the findings were integrated within the proposed model. The adoption of the results investigated interventions needed in three parts of the informal vending industry. Part A of Figure 2 shows the risks related to the physical work environment (which includes the entire surrounding, infrastructure, and tools used by vendors), Part B looks into risks related to the individual vendor’s knowledge, attitude, and behaviour (KAP), and the impact of such behaviour on their health status. Part C investigates the vendor’s health status, which posed questions such as “is the vendor in a good health state”, “do they need further medical assistance”, and “how do we prevent further or future illnesses”. Part A (physical work environment) can directly impact Part C (personal health status), either positively or negatively; however, variables in Part B (workers’ knowledge, attitude, and practices) can have an influence on Part C (human health status). Any gaps identified in Part A, B, or C (Figure 2) should result in control measures being implemented at the point of exposure or risk. The findings relevant to KAP, such as the usage of proper personal protective clothing or equipment (PPE) and handwashing, influence the need for training or reinforcement.

### 3.4. The Proposed Informal Vendors Integrating Healthy Workplace Management Model

**Figure 3:**
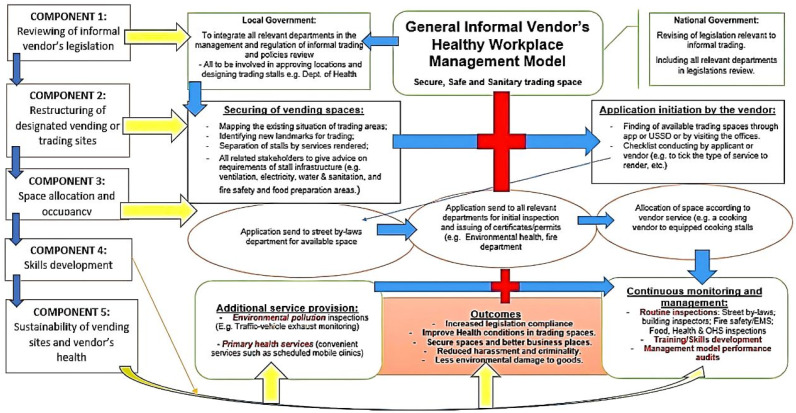
The general informal vendors’ integrated healthy workplace management model.

**Figure 4:**
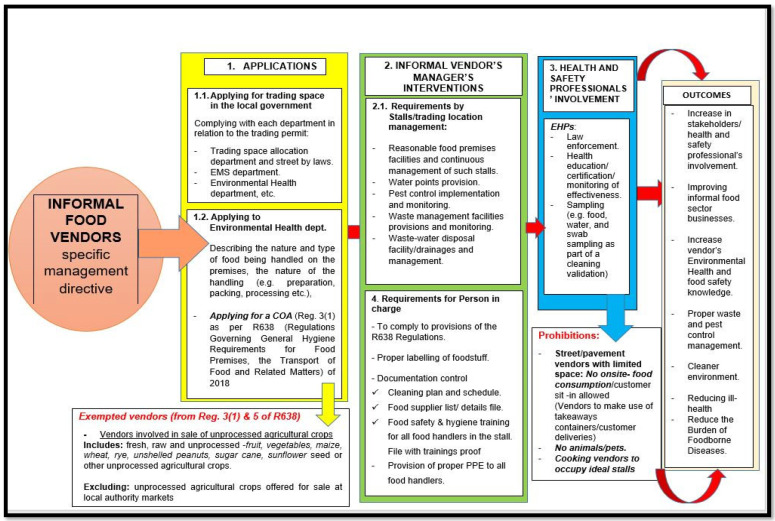
Specific food vendor’s management directive.

This integrated healthy workplace management model includes five main parts which are the reviewing of informal vendor’s legislation, the restructuring of designated vending or trading sites, space allocation and occupancy, vendor’s training and skills development, and, lastly, the sustainability of vending sites and vendors’ health (Table 1). The theme of the proposed integrated management model is to promote the three-“S” secure, safe, and sanitary trading places (Figure 3). The general informal vendors’ model was used to create a specific informal food vendors’ directive (Figure 4). According to the model, there is a need for intervention by all related stakeholders in the management of this trade, thus including looking into the laws and policies guiding vendors, the roles of departments in the management of vendors, and the internal and external structures of stalls. There is a need for the more sustainable management of this trade, with strong relationships between stakeholders and ongoing activities. OHS programmes should themselves be integrated into a bigger piece of work to strengthen the capacity of organizations of informal workers. Furthermore, this may increase the rate of compliance amongst vendors. The formalization of informal vendors, according to Roever (2013), may entail having a registered business and paying more taxes and fees, but it also entails exercising fundamental rights, such as the right to work and earn income without being subjected to harassment, discrimination, or degrading treatment [26].

#### 3.4.1. Component 1: Reviewing of Informal Vendor’s Legislation

When it comes to informal vendors, law enforcement faces difficulties. Currently, the legislative framework for informal trading includes the Constitution of the Republic of South Africa Act (Act 108 of 1996) [27], the Business Act (Act 72 of 1991) [28], and the Municipality’s informal trading by-laws and policy. The vendor’s policies and legislation demand political commitment, a bottom-up approach, and other relevant entities’ involvement such as the DOH, and furthermore, the need for strengthening legislation implementation. Bénit-Gbaffou (2015) reported that the current challenge is that frameworks for street trading are created on a national level but fail to address local problems, such as their dual and frequently incompatible mandates to manage congested streets and encourage economic development and alleviate poverty [5]. Moreover, progressive ideas and policies apply to national policy frameworks, but they rarely address local issues and implementation obstacles in detail [5]. Currently, the legislation of informal vendors is fragmented. In terms of transparency and consistency, there can be a general policy that outlines all standards for informal food premises and the regulation of this industry that every local government can adopt. Perhaps the best course of action would be to also pass a law that specifically addresses informal or street trading. India took a different path than many countries by enacting the Street Vendors Act (Protecting Livelihoods and Regulations on Street Vendors) in 2014 [29]. This Act was enacted to regulate the activities of street vendors in public places and protect their rights [29].

In terms of food vendors, the integration will be required within the Regulations governing general hygienic requirements for food premises, transportation, and related matters (R638) [11]. R638, which is largely enforced by Environmental Health Practitioners (EHPs), applies to places where food is handled. According to regulation R638 [11], a food premise is “a building, structure, stall, or other similar structure, including a caravan, vehicle, stand, or place used for or in connection with the handling of food.” The definition of food premises in Regulations R638 does include informal vendors, but further reading reveals more details about what this type of premise should structurally look like and what type of facilities it should contain in order to be compliant with the regulation. Regulations 5, 6, 7, and 8 of R638 [11] which focus on the facility, internal structure, cooking storage, food handling equipment, and utensils do not clearly cater to most of the current types of informal food trading stalls that vendors occupy. The context in which most informal food vendors operate is unrelated to the provisions of the regulation, which may prevent them from qualifying for the Certificate of Acceptance (CoA) under Regulations 3(1) of the R638 [11]. As a result, applying R638 to this industry would be hampered or complicated. This is a gap or grey area that should be addressed by the law or policymakers.

This separation or minimization from the formal premises’ requirements could include providing the provision of the best available method to an informal vendor in order to avoid different municipalities or law enforcement officers interpreting how they deal with this group.

#### 3.4.2. Component 2: Restructuring of Designated Vending or Trading Sites

Most trading places are currently not conducive workplaces. Formalization requires having a secure vending site in a favourable position in the city [26]. The management model illustrated in Figure 1 is used to clean up the current trading locations and distinguish between conducive and non-conducive ones. Securing vending sites including detailed mapping of the current makers or locations is crucial because often, official planning processes do not show what is happening informally; thus, there is no documented information on the scale and size of vending activities. The mapping should consider the following: numbers of vendors at different times; types of goods sold; location of facilities, e.g., toilets and taps. This mapping might solve the current security and health and safety issues as per this study’s findings. Followed by identifying new market locations, in exceptional cases, it may not be possible to accommodate street vendors on-street, and off-street provision may be the only alternative. However, it must be considered that location is a crucial determinant of a street vendor’s income, and a move of a few meters can drastically reduce their daily earnings. Examples of places that can form part of new vending sites are the rehabilitation of decayed buildings and nearby landmarks such as taxi ranks and malls, which can be designed in a way that will attract the public. Any rearrangement of space should accommodate all existing vendors; otherwise, those excluded will suffer increased hardship and poverty. 

Proper trading market and stall designs will assist in compliance with the by-laws of local government and national legislations, and the expertise of town planning, building control, EHPs, and fire and safety departments are required. Vendors need shade and shelter to protect them and their goods from sun, rain, and dust. The presence of designated sites and the separation of vendors trading stalls by type of services should be achieved. To avoid cross-contamination, the separation of vendor services such as informal hairdressers from food vendors will be vital.

Moreover, this grouping allows for the provision of common facilities and basic services (potable water and electric supplies, storage facilities, central waste storage and collection services, wastewater drainage systems, ablution facilities, vehicle parks, etc.). The processed food stalls shall be designed differently from the unprocessed foodstuff stalls. For cooking food vendors, the provision of extra infrastructure for food vendors thus includes a stainless-steel preparation area, a hand wash basin, wash-up sinks, electricity for cooking equipment, and an extractor fan for cooking vendors (Figure 3 and Figure 4). To ensure the non-interference of street walks, stalls or markets are to be designed in a way that leaves spaces for side walking and parking areas. This will avoid the blocking of pavements and parking spaces and causing congestion for other road users.

#### 3.4.3. Component 3: Space Allocation and Occupancy

Application initiation by the vendor: there is a need to strengthen technical support in the local government in the management of informal trade. The use of e-services (electronic services) should be an important factor in managing informal trade as an alternative or additional channel to the current form, depending on the organization’s capacity. This method of information and communication management makes the process transparent, easier, and manageable. The e-service, provided through an app and USSD (Unstructured Supplementary Service Data), is one of the most reliable communication technologies available to provide to low-income individuals or those without smartphones. This service should reflect the newly mapped vending sites or markets. The potential vendors should be able to see how many stalls are occupied at that designated market and the available trading stalls. The application should include the detailed nature of the trade or the type of service they will be rendered to achieve the separation of food vendors from services, such as hairdressing services. For street or outdoor trading, the application should be sent to the street by-laws department or applied for in person with the stall address and stall number, and they should be issued with any certification or proof of application before the other certification of other departments. If vendors are planning on selling or hawking food, then they will need to apply for a hawking business trading license as well as a certificate of acceptability. However, to ensure better compliance, skills development such as training for food vendors should be conducted with proof of training filed prior to getting the final trading permit. The permit should be able to be found online using the identification number and permit number, showing the full names of the vendor, the address of the stall, and the type of service rendered. the institution should be able to electronically produce the current database of the registered vendors at any time of need.

#### 3.4.4. Component 4: Vendors’ Skills Development

In various local government by-laws, it is outlined that a workshop covering every aspect of street trading in detail must be attended by those who have been given a trading opportunity within six months of receiving the authorization to trade [6]. Most vendors’ low level of education makes training difficult. As a result, training industry personnel and street food vendors must take very different approaches. To achieve this, training materials addressing simple messages must be developed and implemented for the latter group. Furthermore, the training can be in their mother tongue so that they can comprehend the training content. The study’s findings demonstrate the need for and importance of repeated and measurable training programs. Examples of training needed as per the study’s findings include proper handwashing practice, the wearing of protective clothing, knowledge of air pollution, and other work-related risk factors and their impact on health. All health-related campaigns should target groups, such as informal vendors, in a way that addresses both preventative and curative measures at the same time.

The health and safety promotion campaigns should be continuous, and proper monitoring of the impact of the campaigns should be implemented. The education should also include the impact of cooking exposures, ambient air pollution, and the impact of general hygiene on health. Food handlers should receive training in safe food preparation and handling techniques, occupational hazards, as well as excellent hygiene habits. It is a crucial part of any strategy to improve the food quality and the safety of street vendors, to the extent that is practical under local street vending conditions. This should ideally be conducted concurrently with licensing, but periodic education and training sessions are strongly recommended. Appropriate authorities may develop food handler training programs and materials based on the concepts presented in this text and other publications and tailored to local foods, conditions, and practices. Health promotion and disease prevention programs should empower individuals to make healthier choices and reduce their risk of disease and disability.

#### 3.4.5. Component 5: Sustainability of Vending Sites and Vendor’s Health

In the continuous monitoring and additional services provision: The current study showed that there is an impact on the usage of dirty cooking fuels and the health of vendors. Masuku and Nzewi (2021) stated that we keep in mind that informal food enterprises occasionally use traditional sources of energy, such as wood and charcoal, not because they lack access to electricity, but rather because of the type of product they are preparing [30]. The lack of access to modern and efficient energy sources has a negative impact on the user’s health, and using wood and charcoal can cause respiratory diseases, moreover, there is a safety aspect of the usage of an open fire, as various markets have burnt over the years, hence the need for the monitoring and empowerment of vendors. Health professionals can conduct OHS education without the use of expensive equipment, making it highly possible to increase in implementation. The DOH and health-related non-governmental organizations should stop working in silos and start working together. 

Environmental management and OHS services: Market occupational health risk assessment should be implemented as an ongoing program. There is a need to rank street vending operations according to risk, such as identifying the critical practices of specific street vending operations and assisting in the risk classification of operations. Ambient air pollution was reported as a risk factor in various street vendor studies, with most sources of exposure being vehicle emissions; vehicle exhaust testing and law enforcement in traffic-congested places, such as urban areas, will contribute to the improvement of air quality. Vehicle emission tests can be performed regularly.

Environmental health: The environmental health sampling programs should include food and water and swap sampling at informal vendor premises for the continuous analysis of the conditions and to monitor improvements.

Primary health care: The primary health care directorate should investigate the provision of mobile clinic services closer to trading markets, or provide scheduled drive campaigns to improve the health status of informal vendors and prolong life. This can include programs such as general check-ups, respiratory health care assessments, detecting health issues as early as possible, and responding appropriately to avoid the increasing prevalence of respiratory diseases associated with being a street vendor. Furthermore, because women dominate this industry and have been identified as being affected, it is suggested that special attention be paid to them, particularly in regard to working as a vendor during pregnancy and exposure to such pollutants and their impact on the health of the woman and the unborn child. The vendors should also be educated on the importance of being vigilant in monitoring personal health symptoms and contacting their employers or managers if they begin to feel ill. These programs will be improved with local primary health service facilities that record patients’ job types on files or welcome registers, which can later be used to find factors contributing to illnesses in the country such as the type of occupation.

Management model performance audits: There should be a directorate within the government that will manage the system and interaction between the various departments. With that, a clear development of key performance areas for the improvement of this trade should be outlined. Furthermore, this directorate should use the communicated reports of activities conducted and map out the performance of the model.

## 4. Discussion

The International Labour Organization’s (ILO) recommendations for integrating OHS services in developing countries’ public health systems include the prevention and early detection of illnesses and occupational injuries [31]. The WHO and NIOSH institutions also advocate for policies that promote and sustain health, which incorporate employees’ health into other policies. However, there is also a need for vendors to play their part in abiding by legislation and policies designed for their trade. This will assist in creating positive law enforcement grounds in cities. In South African policy making, the informal sector continues to be substantially marginalized [32]. The legislation or regulation of informal vendors’ activities continues to be fragmented, leading to different types of interpretation in different municipalities. In addition, current findings among informal suppliers in South Africa and other countries demonstrated that the health of salespeople is affected by working conditions, hence the need for new management strategies. The burden of respiratory diseases could lead to an increase in healthcare costs that could have an impact on the health of the entire population [33]. 

To the best of our knowledge, the currently developed informal vendor’s models or reported management strategies are based on the informal sector economy and safer food, nutrition, and hygiene. The majority of the studies’ models are based on data from a limited scientific assessment of this trade, such as air pollution sampling. Even while existing models are built on similar conceptual foundations and use similar modelling procedures, it can be challenging to compare them due to variations in datasets and validation methodologies [34]. The Bank for International Settlement (2010) noted that applying models to exposures that are different from those that were observed during the creation of the model or using models with incomplete or undocumented model elements can make it difficult for model users to undertake accurate evaluations of models [34].

However, the proposed model followed the requirements of the WHO’s healthy workplace requirements. In addition, the model has been explained in detail to facilitate implementation; moreover, it has applied the evidence found in the field and used guidance from relevant legislation. The difference with this model is that it examined institutional interventions focused on the workplace, interventions focused on the KAP of the informal vendors, and separately, the personal health of the vendors. It integrated environmental and OHS into the public health system, thus including primary health care services in the management of informal vendors. Furthermore, it fits the description of a community model which is carried out by governmental institutions [24]. The OHS services will be provided within the community health programs which will be delivered through already-developed networks. It is simple to include into the current framework. 

Health professionals should receive assistance from central or regional centers and increase capacity-building training on occupational health. This integrated management includes the strengthening of informal vendor organizations, government institutions, and the support of all involved to ensure the livelihood of informal vendors and to ensure a long-lasting and sustainable solution to the problems faced by informal vendors.

## 5. Conclusions

The study findings showed the association between upper respiratory symptoms and the type of work location, type of fuel used, duration of work, use of protective clothing, and hand hygiene amongst informal vendors. Moreover, vendors were extremely exposed to environmental health hazards due to a lack of infrastructure. In conclusion, there is a lack of studies, frameworks, or models in SA about the occupational risks and management opportunities for informal vendors. This proposed evidence-based model was informed by the current study and the current requirements of the legislation related to food premises and other health legislations, thus integrating the occupational health and safety of informal vendors into the public health system. When raising awareness about ill health, including air-pollution-related ill-health, the government and non-governmental organizations must consider informal vendors as a target group. There is a need for collaboration among departments, local government heads, the community, and the associations of informal vendors. Informal vendors should be recognized and, where possible, included in urban development programs. The provision of such infrastructure and services is typically very expensive, and most governments lack the resources to implement this strategy on a large scale, hence the need for government and non-government stakeholder involvement. As per ILO, the drawback of this type of model is that it may overload primary healthcare facilities in densely populated locations [24]. However, local government and public health entities should trial this model or components of the model for the better inclusion of informal vendors into the health system. Furthermore, the implementation of the model should be in phases to reduce overloading to any party involved. For example, implementing components 1 and 2 as Phase 1 includes sitting down as a government department to revise and integrate policies and mapping out the city to identify trading sites. Then, the other part of the components will follow through. Key success factors behind this type of model implementation were found to be political will, leadership, social dialogue, and partnership [19]. The local governments struggling with the management of informal vendors have a lot of work to do (planning and various programs implementation programs).

## Figures and Tables

**Table 1:** Description of the components as per items listed on the informal vendors’ integrated management model.

No	Component	Item on the Model
1.	Reviewing of informal vendor’s legislation	National and local government roles.
2.	Restructuring of designated vending or trading sites	Securing vending sites; redesigning stalls
3.	Space allocation and occupancy	Application initiation by the vendor and stall occupancy.
4.	Skills development	Continuous monitoring and management (inspections; training and skills development)
5.	Sustainability of vending sites and vendor’s health	Continuous monitoring and management (inspections; management model performance audit) and additional service provision

## Data Availability

All data in this study were provided in the main manuscript.
